# Improving Probiotic Strawberry Dairy Beverages with High-Intensity Ultrasound: Syneresis, Fatty Acids, and Sensory Insights

**DOI:** 10.3390/foods14040616

**Published:** 2025-02-13

**Authors:** Amanda Gouveia Mizuta, Eloize da Silva Alves, Jaqueline Ferreira Silva, Paula Gimenez Milani Fernandes, Silvio Claudio da Costa, Carlos Eduardo Barão, Tatiana Colombo Pimentel, Andresa Carla Feihrmann, Benício Alves de Abreu Filho, Suelen Siqueira dos Santos, Grasiele Scaramal Madrona

**Affiliations:** 1 Headquarters Campus, Postgraduate Program in Food Science, State University of Maringá (UEM), Maringa 87020-900, Brazil; amandagmizuta@gmail.com (A.G.M.); eloizeetaus@gmail.com (E.d.S.A.); jaquelinesferreirasilva@gmail.com (J.F.S.); pgmfernandes2@uem.br (P.G.M.F.); sccosta@uem.br (S.C.d.C.); andresafeihrmann@gmail.com (A.C.F.); baafilho@uem.br (B.A.d.A.F.); 2 Federal Institute of Paraná (IFPR), Food Production, Paranavaí Campus, Paranavaí 87703-536, Brazil; carlos.barao@ifpr.edu.br; 3 Jandaia do Sul Campus, Food Engineering, Federal University of Paraná (UFPR), Jandaia do Sul 86900-000, Brazil; suelensiqueira@ufpr.br

**Keywords:** syneresis, milk beverages, consistency, consumer acceptance

## Abstract

Consumer acceptance of milk beverages as probiotic beverages is directly linked to their sensory qualities, such as flavor, consistency, visual appearance, and mouthfeel. Overall, products that exhibit syneresis are often viewed as inferior. Thus, this study was conducted to investigate the effects of high-intensity ultrasound on the production of probiotic strawberry beverages, aiming primarily to stabilize the beverage by reducing syneresis and improving sensory properties without compromising the viability of probiotic microorganisms. The effects of the ultrasound processing time (2.5, 5, 7.5, and 10 min) on the physical, chemical, and sensory properties of the beverages were analyzed. Ultrasound was applied using a 750-wW ultrasonic processor (Cole-Parmer^®^, 750 W, Vernon Hills, IL, USA) at 40% amplitude, consuming 300 W and resulting in an acoustic power density of 1.2 W/mL. The results indicate that ultrasound significantly influenced the syneresis of the samples, with intermediate times (5 and 7.5 min) demonstrating lower liquid separation. Notably, the U7.5 treatment exhibited syneresis values of 52.06% ± 2.14, 60.75% ± 2.33, and 61.17% ± 1.90 at days 1, 14, and 28, respectively, corresponding to reductions of approximately 18%, 12%, and 11% compared to the control (63.43% ± 0.93, 68.81% ± 0.56, and 68.59% ± 0.10, respectively). The fatty acid composition showed changes according to storage time. Notably, palmitic acid (C16:0) concentrations were above 30 g/100 mL, and the ω6/ω3 ratio ranged from 5.92 to 7.47, falling within the recommended dietary values. Ultrasound also reduced the amount of sucrose in the samples, which may benefit the growth of probiotic microorganisms. In terms of sensory analysis, the ultrasound-treated samples (2.5 to 7.5 min) were preferred by the evaluators compared to the control sample. Furthermore, ultrasound treatment did not result in the inactivation of probiotics, supporting its potential for enhancing probiotic beverage quality. Thus, high-intensity ultrasound proved to be a promising technology for enhancing the quality of probiotic strawberry beverages by reducing syneresis, affecting fatty acid composition, and improving sensory characteristics. This may open up new opportunities in the food industry for more appealing and healthier probiotic products.

## 1. Introduction

Research indicates that intestinal health is closely linked to the rest of the body and can significantly impact skin, immunity, and cognitive functions. Many consumers recognize the vital role of maintaining intestinal health for overall well-being and adhere to diets supporting the microbiota, such as the consumption of prebiotics and probiotics, which have become a burgeoning trend, offering new opportunities for the development of products that support a healthy intestinal microbiota and potentially improve health. Traditionally, fermented dairy products have been the primary source of probiotics [1,2,3].

Consumers understand the health-promoting role of probiotics. According to the research by Cosme et al. [1], at least 54% of consumers globally know that probiotics promote good digestive health. This awareness has driven increased demand for and sales of probiotics incorporated into foods worldwide, forming the largest segment of functional foods [4]. Consequently, there is a need for technological research to improve production processes and the final products.

High-intensity ultrasound (HIUS) has emerged as a potential technology for adoption in the dairy industry. Parameters such as pH, characteristic fat globules, stability, or composition alteration are crucial for dairy processors. Improving these parameters in dairy beverages has been a common goal among researchers and processors. Physicochemical parameters in dairy products are essential for quality acceptance, and high-intensity ultrasound has proven its potential to provide positive effects in relation to these aspects. Therefore, HIUS holds significant prospects in the food processing industry for improving process efficiency [5]. Also, the primary application of ultrasound in this study aims to stabilize the probiotic strawberry beverage, reducing syneresis and improving sensory properties without compromising the viability of probiotic microorganisms. This approach is consistent with the findings of Mizuta et al. [6], who observed that ultrasound treatments for times of 2.5, 5, 7.5, and 10 min did not result in the inactivation of probiotics.

A study on yogurt with the application of high-amplitude ultrasound demonstrated a satisfactory homogenization effect compared to conventional homogenization. It also increased viscosity and the water retention capacity, reduced syneresis, improved gel strength and firmness by enhancing whey protein coagulation properties, and decreased the fermentation time by improving lactose hydrolysis and stimulating probiotic bacteria [7].

Several mechanisms are suggested to describe the role of ultrasound in inducing the fermentation process. The promoting effect of ultrasound on fermentation is mainly attributed to the formation of repairable damage to bacterial cell membranes. This damage enhances membrane permeability, creating channels for the transport of essential nutrients and the removal of toxic substances from microbial cells. This mechanism is known as “sonoporation” [8]. Additionally, cavitation forces associated with ultrasound can increase reaction rates. Ultrasound treatments have been reported to result in structural modifications in milk gels. Nguyen and Anema [9] observed an increase in gel strength along with a reduction in the gelation time of fermented cow’s milk after pre-treatment with US (22.5 kHz and 50 W) due to increased intermolecular interactions between the milk proteins, leading to a dense gel network.

Consumer preference is based on quality characteristics, such as color, flavor, aroma, and texture, as well as nutritional characteristics. In this context, this study aimed to investigate the impact of the use of ultrasound processing for different periods of time in strawberry probiotic fermented beverages, evaluating the profile of sugars, the profile of fatty acids, and the sensory attributes.

## 2. Materials and Methods

### 2.1. Probiotic Culture and Ingredients

A Christian Hansen^®^ lyophilized commercial culture of Lacticaseibacillus casei (LC01) was used. Strawberry pulp, milk, and sugar were commercially obtained in Maringá, PR, Brazil. Whey powder (SW1108) was obtained directly from the Alibra S.A. industry, Brazil.

### 2.2. Preparation of Strawberry-Flavored Probiotic Fermented Beverages

The fermented beverages were produced according to the method described by Guimarães et al. [10], with modifications. Five formulations of fermented beverages were processed: a control (without ultrasound), ultrasound (U)2.5 (2.5 min), U5.0 (5 min), U7.5 (7.5 min), and U10.0 (10 min). For the production of the beverages, whole pasteurized cow’s milk (30 g 100 g^−1^), demerara sugar (8 g 100 g^−1^), whey powder (6 g 100 g^−1^), strawberry pulp (21 g 100 g^−1^), and water (35 g 100 g^−1^) were used. First, the whey powder was dissolved in milk and mixed with water, strawberry pulp, and sugar. The mixture was homogenized using a mixer (New Lab^®^, NL-150-01, Piracicaba, SP, Brazil) at 18,000 rpm until complete homogenization was achieved. Then, all the formulations were pasteurized (65 °C, 30 min) using a water bath (Marconi^®^,Piracicaba, SP, Brazil) and cooled down (25 °C). Then, 0.1 g 100 g^−1^ of L. casei was added to all the beverages and homogenized. The ultrasound-treated beverages were processed in an ultrasonic processor (Cole-Parmer^®^, 750 Ws, Vernon Hills, IL, USA) using 750 W at 40% amplitude for the specific times, with 30 s on and 10 s off. The samples (250 mL) were treated in beakers, and the probe was dipped 10 mm into the sample. The power consumed was 300 W (40% amplitude), resulting in an acoustic power density of 1.2 W/mL. The beakers with the samples were kept on ice during sonication, and the temperature was measured and controlled throughout the process using a digital thermometer (HI98509 Checktemp^®^1, Barueri, SP, Brazil). The final temperature for all ultrasound treatments was approximately 28 ± 2 °C. Afterwards, all the beverages were incubated at 37 °C until they reached pH 4.6. At the end of fermentation, the beverages were stored in a refrigerator at a controlled temperature of 7 °C for 28 days [11].

### 2.3. Measurement of Syneresis

Syneresis of the probiotic fermented milks was determined as described by Keogh and O’Kennedy [12], with suitable modifications. The probiotic beverages were centrifuged (Centribio) at 2500 rpm for 10 min at 4 °C, and then the supernatant was collected and weighed. The syneresis percentage was determined using Equation (1):

Syneresis (%) = [(Weight of supernatant (g))/(Weight of probiotic fermented milks (g))] × 100(1)

The syneresis was evaluated on days 1, 14, and 28 of storage.

### 2.4. Scanning Electron Microscopy (SEM)

The samples were lyophilized in lyophilizer Alpha 1-2 LD Plus (model 101522, Osterode, Germany) under conditions of −54 °C and 0.021 mbar until a constant weight was obtained after drying. The dried samples were prepared on carbon tape and metalized with a thin layer of gold (≈50 nm) on the surface in a metallizer (SCD 050, Bal-Tec, Obermattstrasse, Switzerland). This was followed by evaluation in a scanning electron microscope (Fisher Scientific-FEI, model Quanta 50, Brno, Czech Republic) under 5000× magnification, with an acceleration voltage of 20 kV. Scanning electron microscopy (SEM) was performed on days 1 and 28.

### 2.5. Fatty Acid Profile by GC/MS

The fatty acid content was evaluated on days 1 and 28. Total lipids were transmethylated as described by Hartman and Lago [13]. The fatty acid methyl esters (FAMEs) were separated on a 7890A Agilent gas chromatograph, coupled to a mass detector (Agilent 5975C), using an RT-x wax polyethylene glycol column (30 m in length × 0.25 mm internal diameter). Hydrogen was used as the carrier gas, and the sample injection volume was 1 µL at a split ratio of 1:50. The injector and detector temperature was 250 °C, while the column temperature was 80 °C for 2 min, which was then increased to 235 °C at a rate of 4 °C/min, remaining at this temperature for 10 min. The identification of fatty acids was performed using the NIST library (MS Search version 2.0), and the quantification was based on the relative area of the methyl ester internal standard of C23:0 (methyl tricosanoate). The results were expressed in g/100 g of fatty acids.

The nutritional quality of the lipid content was determined considering the composition of fatty acids, based on the literature that determines the relationship between representative fatty acids and those that play an important role in the health of individuals. Therefore, to determine the lipid nutritional value, the relationships between individual fatty acids or their groups are indicated [14]. The defined relationships encompass the sum of saturated fatty acids (ΣSFA), the sum of monounsaturated fatty acids (ΣMUFA), the sum of polyunsaturated fatty acids (ΣPUFA), the sum of the groups of omega-3 fatty acids (Σn-3) and omega-6 fatty acids (Σn-6), the ratio of omega-6/omega-3 fatty acids (Σn-6/Σn-3), and the ΣPUFA/ΣSFA ratio [5,13].

### 2.6. Quantification of Sugars by HPLC

The quantification of sugars was evaluated on days 1, 14, and 28 of storage, following the method described by Dacome et al. [15], with modifications. The quantification was performed using external standards for fructose, glucose, galactose, sucrose, and lactose (1 mg/mL). The mobile phase consisted of acetonitrile/water (80:20, *v*/*v*). For sample preparation, 5 mL of the liquid sample was diluted with water in a 100 mL volumetric flask. From this solution, 2 mL was further diluted with 8 mL of acetonitrile. The resulting solution was filtered and injected into a CG model 480-C (São Paulo, SP, Brazil) liquid chromatograph equipped with an NH2–silica column (125.0 mm × 4.6 mm), operated isocratically at a flow rate of 0.5 mL/min at room temperature under a pressure of 33 bar and monitored with a Waters 410 DRI detector.

### 2.7. Sensory Analysis

Sensory analysis was evaluated on the first day and was performed shortly after manufacture at the Sensory Analysis Laboratory at the State University of Maringá. An acceptance test was applied using a 9-point hedonic scale, as described by Meilgaard et al. [16]: 1—dislike extremely, 2—dislike very much, 3—dislike moderately, 4—dislike slightly, 5—neutral, 6—like slightly, 7—like moderately, 8—like very much, and 9—like exceptionally. The color, aroma, texture, flavor attributes, and overall acceptance of the formulations were evaluated using a team of 100 tasters and potential consumers. For the evaluation, 20 mL samples were presented to tasters in a balanced way in disposable white plastic containers coded with random three-digit numbers. The project was approved by the Ethics Committee of UEM under number 52725921.1.0000.0104. The acceptance index (AI) was calculated by taking the average score obtained in overall acceptance, dividing it by the highest score, and multiplying by 100.

### 2.8. Statistical Analysis

All analyses were conducted in triplicate. Data were analyzed using two-way ANOVA (analysis of variance), followed by Tukey’s mean comparison test, using Sisvar Software version 5.6 and a significance level of *p* < 0.05.

## 3. Results and Discussion

### 3.1. Syneresis

Syneresis is an unsatisfactory phenomenon that affects the quality and consumer acceptance of dairy beverages, occurring with the elimination of whey or water from the gel matrix formed during beverage processing [17,18]. It can impact the sensory quality of the beverage in various ways, such as reducing firmness and viscosity and giving the appearance of lumps, which can be perceived in the mouth [19]. In the case of this article, beverages treated for intermediate times (5 and 7.5 min) achieved satisfactory results compared to the control and probiotic beverages treated for short (2.5 min) and long (10 min) times.

The syneresis results of the control beverage and beverages subjected to HIUS for different times are presented in Table 1. The effect of an ultrasound processing time of up to 7.5 min was more pronounced than the effect of the sample storage time. The U7.5 treatment exhibited syneresis reductions at days 1, 14, and 28 of approximately 18%, 12%, and 11%, respectively, compared to the control. As shown in the results, during the storage period from day 01 to day 14, the samples were not stable, indicating a significant difference (*p* < 0.05) in all samples. From day 14 to day 28, there was no significant difference in this parameter (*p* > 0.05).

On days 01, 14, and 28, except for the U10 sample on day 01, all the beverages subjected to HIUS treatment showed a significant difference (*p* < 0.05) compared to the control. The U2.5 and U10 samples did not differ from each other, meaning that intermediate times such as 5.0 and 7.5 min had a more significant influence on this parameter than short (2.5 min) and long (10 min) times.

When comparing the syneresis results presented in this study with the instrumental texture data obtained by Mizuta et al. [6], a reduction in firmness and consistency in the samples was observed during the storage period from day 1 to day 14. However, from the 14th day of storage onwards, the samples demonstrated stability (*p* > 0.05), confirming the validity of the syneresis results presented in Table 1.

Furthermore, when analyzing the samples on the same storage days (1, 14, and 28), we observed that the probiotic beverages treated with high-intensity ultrasound for intermediate durations of 5 and 7.5 min (U5.0 and U7.5) demonstrated satisfactory firmness and consistency compared to the control sample. Specifically, firmness values were 29.09 g and 28.73 g on day 1, 27.66 g and 27.85 g on day 14, and 27.85 g and 27.74 g on day 28. Consistency values were 164.18 gs and 162.10 gs on day 1, 157.05 gs and 158.21 gs on day 14, and 157.90 gs and 156.82 gs on day 28. The control sample showed a firmness of 26.81 g and consistency of 151.07 gs on day 1, firmness of 26.88 g and consistency of 151.95 gs on day 14, and firmness of 27.07 g and consistency of 152.99 gs on day 28, reinforcing the validity of the syneresis results obtained in this study [6].

### 3.2. Scanning Electron Microscopy

**Figure 1:**
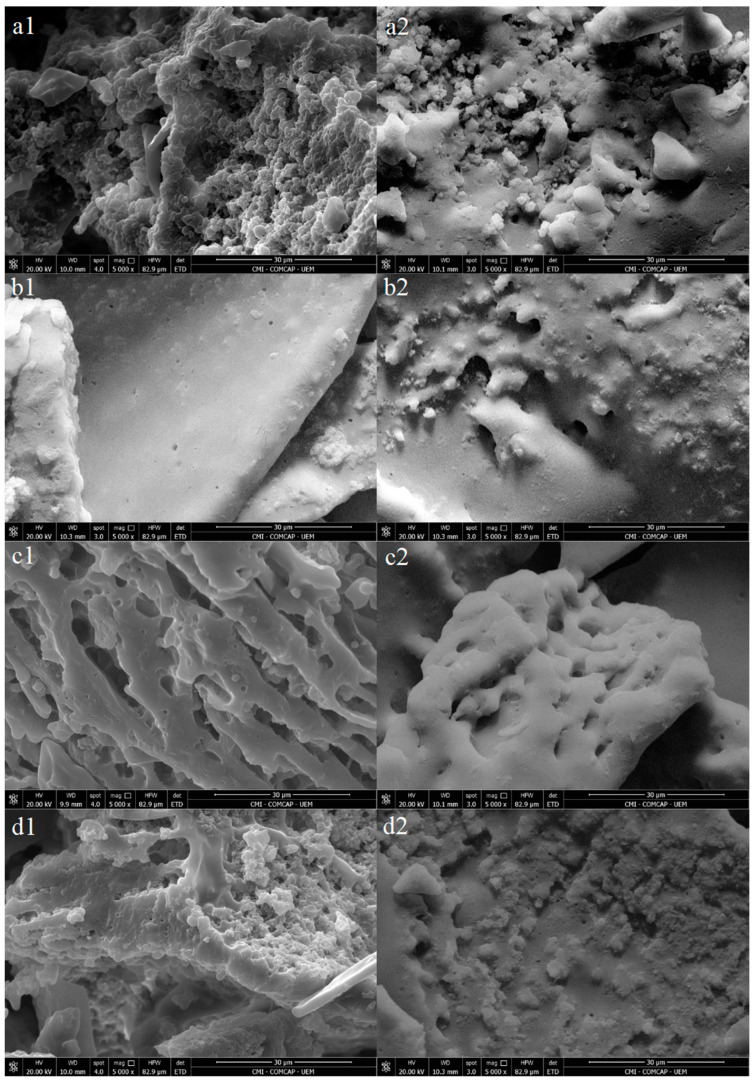
Scanning electron microscopy of the probiotic drinks (control, U2.5, U5.0, U7.5) on days 1 and 28: (**a1**) control—day 1; (**a2**) Control—day 28; (**b1**) U2.5—day 1; (**b2**) U2.5—day 28; (**c1**) U5.0—day1; (**c2**) U5.0—day 28; (**d1**) 7.5—day1; (**d2**) day 28.

Figure 1 shows that beverages treated with high-intensity ultrasound (Figure 1—b1,b2,c1,c2,d1,d2) exhibited a more uniform and smoother surface than the control sample (Figure 1—a1,a2) both on day 1 and day 28. These images highlight that the application of high-intensity ultrasound in probiotic beverages affects their structure.

The application of HIUS in milk and dairy beverages has demonstrated advantages in physicochemical quality parameters. Carrillo-Lopez et al. [5] mention the effect of HIUS on various types of milk, including buffalo milk, sheep milk, supplemented milk, and bovine milk. In their study, the positive effects of HIUS were confirmed, emphasizing improvements in fat globule size, fat crystal size, fat droplets, and other milk particles. These modifications in beverage structure resulted in physical benefits, such as increased gel strength and hardness, accelerated gel formation, the distribution of fat into smaller particles, and homogenization of the beverage.

In juices, different HIUS treatment times resulted in cell ruptures and the release of intracellular components, making the juice more homogeneous and indicating a direct influence of ultrasound on the integrity of juice cells [20]. Thus, in probiotic beverages, such as in the present study, the use of HIUS promoted a more uniform and smoother surface, highlighting microstructural alterations. These results suggest that HIUS is an effective technique for homogenizing the structure of liquid products, with potentially significant impacts on the quality and characteristics of these products. The present research emphasizes the potential of HIUS as a promising tool to enhance the physical quality of dairy products.

### 3.3. Fatty Acid Composition

Through the fatty acid profile (Table 2), it was noted that palmitic acid (C16:0) had a concentration greater than 30 g/100 mL, with the highest content among the saturated fatty acids identified. In addition, the presence of capric acid (C10:0), lauric acid (C12:0), myristic acid (C14:0), pentadecanoic acid (C15:0), margaric acid (C17:0), and stearic acid (C18:0) was included for all formulations. The samples also exhibited unsaturated fatty acids, such as linoleic (C18:2n-6), linolenic (C18:3n-3), oleic (C18:1n-9), and arachidonic (C20:4n-6) acids.

Short-chain fatty acids have 4 to 6 carbons, medium-chain fatty acids have 8 to 12 carbons, and long-chain fatty acids have more than 12 carbons. No short-chain fatty acids were found. It is important to highlight that these changes in relation to the fatty acid chains can be influenced by the power used in the ultrasound. For example, Guimarães et al. [21] evaluated a prebiotic drink made from whey and soursop and observed that the application of 400 W ultrasound promoted lower production of volatile compounds and short-chain fatty acid content; however, powers of 200 and 600 W made no difference (*p* > 0.05) compared with the conventional treatment.

Saturated medium-chain and long-chain fatty acids, such as caprylic, capric, and lauric acid, decreased during the 28-day storage. Myristic and palmitic acids increased in the U2.5 sample during storage. Margaric acid increased in the HIUS-processed samples during the 28-day storage, and stearic acid increased during storage in all samples.

Medium-chain and long-chain monounsaturated and polyunsaturated fatty acids exhibited different behaviors during storage (from day one to day twenty-eight) for each sample. From day 01 to 28, methyl tetradecanoate (C15:2) decreased only in the control, and in the HIUS-treated samples, it remained stable. The quantity of linoleic acid, a functional lipid [22], increased only in the U5.0 sample and decreased in the other samples (*p* < 0.05) over time. Oleic acid increased from day 01 to day 28 in all samples (*p* < 0.05) except the U10 sample. Linolenic acid, with potentially beneficial biological effects, including anticarcinogenic, antiatherogenic, antidiabetic, and immunological stimulation [23], remained stable from day 01 to day 28 in the control and treatments for 2.5 and 5 min, unlike the 7.5 and 10 min treatments, which showed a reduction in this acid during storage. Elaidic acid increased with storage time (*p* < 0.05) except in the U10 sample. Arachidonic acid remained stable, showing no changes during storage (*p* > 0.05) in any sample.

Changes in the fatty acid composition of fermented dairy products are influenced by processing conditions and lipolytic activity during storage [24]. Previous research indicates that ultrasound significantly alters the fatty acid proportions in probiotic yogurt during storage. This phenomenon is associated with oxidation induced by acoustic cavitation, shear forces, or lipolysis, which results from increased lipase and esterase activity triggered by ultrasound application [25,26].

The results show that the ∑PUFA/∑SFA ratio found in the samples was below the expected values, as values below 0.45 are considered unsatisfactory. Milk fat is almost the sole source of butyric acid (4:0), conjugated linoleic acid (CLA), and branched-chain fatty acids in the human diet. Although they represent only a small percentage of milk fat, small quantities can still be biologically relevant, either individually or within the context of the milk matrix [27].

In functional dairy products, polyunsaturated fatty acids (PUFAs) stand out for their benefits in reducing the risk of chronic diseases [19]. The reduction in fatty acids in the samples may have occurred due to probiotic cultures consuming them through the lipolysis of fat micelles [28].

According to World Health Organization (WHO) and Food and Agriculture Organization of the United Nations (FAO) [29], a ratio of 5:1 to 10:1 is recommended to have a balanced diet in terms of the omega-6/omega-3 ratio; so, all the studied samples were in accordance with this, ranging from 5.918 to 7.474.

### 3.4. Quantification of Sugars

HIUS can be used to break particles and alter compound structures. Probiotic dairy products treated with ultrasound may exhibit higher probiotic survival, increased fermentative activity, and greater peptide production. These improvements are related to significant changes in the environment, such as enzyme release and carbohydrate hydrolysis [30].

As is evident in Table 3, the control sample, which did not undergo ultrasonic treatment, showed a higher amount of sucrose, while all the ultrasound-treated samples had a lower sucrose content on day 01 (*p* < 0.05). This reduction could be attributed to ultrasound breaking down the sucrose molecules. Additionally, comparing these results with those obtained by Mizuta et al. [6], who assessed the viability of probiotic microorganisms over a storage period of 1 to 28 days, it is observed that ultrasound-treated beverages achieved higher viability than the control, indicating that the breakdown of the sucrose molecules favors the growth of probiotic microorganisms. The same analysis can be applied to lactose (a sugar molecule present in milk), as the control significantly differed (*p* < 0.05) from the ultrasound-treated samples.

Fructose, in general, yielded consistent results for all samples, remaining stable from day 01 to 14 and decreasing from day 14 to day 28 (*p* < 0.05). Fructose is a sugar naturally present in foods such as fruits and vegetables. Some probiotic microorganisms, like lactic acid bacteria, can metabolize this sugar as an energy source. This is crucial, as fructose metabolism can play a pivotal role in the survival and growth of probiotic strains. Therefore, it is plausible that probiotic microorganisms utilized fructose as an energy source, a phenomenon already reported in the literature [31].

### 3.5. Sensory Analysis

**Figure 2:**
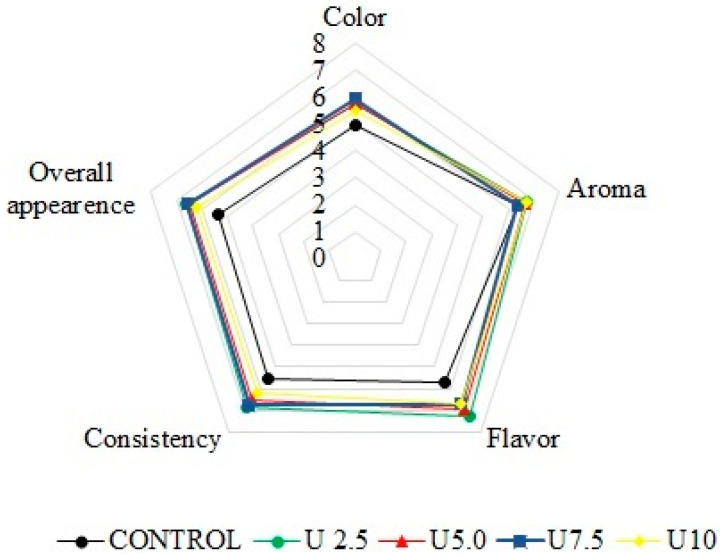
Acceptance indexes of sensory attributes on day 1.

Consumers seek safe, healthy, and flavorful foods, and functional products (probiotics) align with this food consumption trend for the coming years. Figure 2 presents the results obtained in the sensory analysis of the research. In all the evaluated parameters, except for aroma, there was a significant difference (*p* < 0.05) between the control sample and the samples treated with high-intensity ultrasound for different periods of time. The treated samples scored higher (*p* < 0.05) in all attributes. Regarding purchase intention, samples U2.5, U5.0, and U7.5 showed better intentions. It is noteworthy that the highest acceptance indexes were 73% for U2.5, 71% for U5.0, and 72% for U7.5, indicating that the U2.5 sample, besides having a shorter processing time, obtained the highest acceptance index.

**Figure 3:**
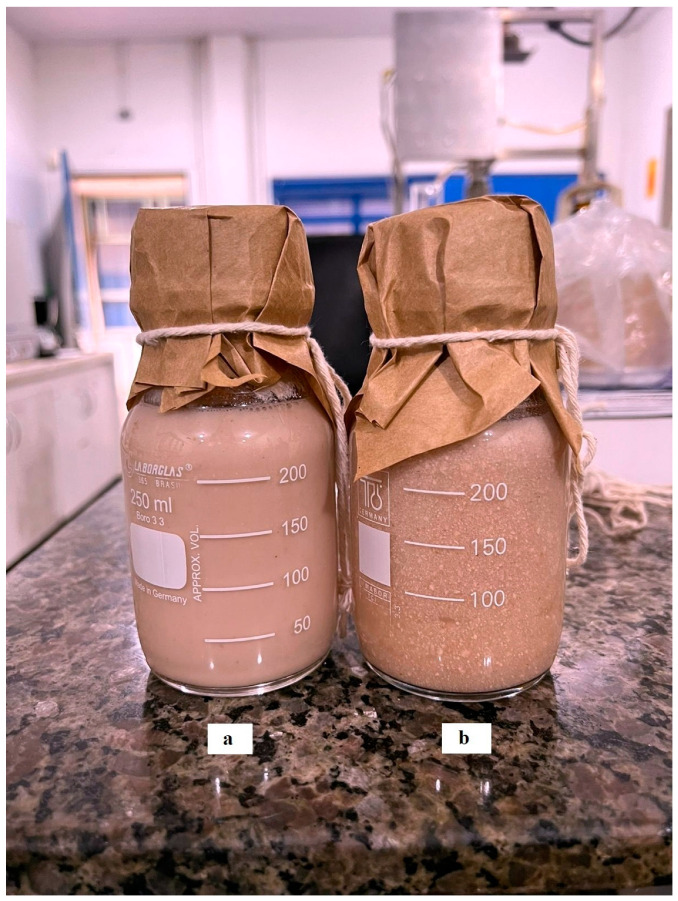
(**a**) Ultrasound-treated (U7.5) and (**b**) non-treated probiotic beverages.

Color is the first sensory attribute observed by the consumer of a food product and is therefore fundamental. The influence of the structure and composition of yogurts on their taste and aroma has been the focus of many studies, as the structure of a product can affect the transport of aromatic compounds to the upper space of the product [32]. The consistency and appearance of the samples treated with ultrasound (U2.5, U5.0, U7.5, U10) achieved significantly higher scores (*p* < 0.05) when compared to the control sample, which corroborates the syneresis results (Table 1) and demonstrates that these parameters are fundamental to consumer choices. Figure 3 shows non-treated and ultrasound-treated (U7.5) probiotic beverages; a lot of precipitates can be observed in the non-treated beverage.

Also, the results obtained for the sensory parameter of consistency were consistent with the texture and rheology results obtained by Mizuta et al. [6], who applied high-intensity ultrasound (HIUS) with different processing times (2.5, 5, 7.5, and 10 min) to evaluate textural properties, demonstrating that samples treated with high-intensity ultrasound are more consistent, an important parameter for dairy beverages. The aroma parameter did not show a significant difference, indicating that the visually significant difference in structure did not interfere with the product’s aroma. Both the control and the treated samples exhibited similar behavior in this parameter.

## 4. Conclusions

HIUS plays a crucial role in stabilizing probiotic beverages over time. Results of the syneresis analysis highlighted the significant influence of ultrasound in enhancing beverage stability over time, especially after day 14 of storage and for intermediate ultrasound treatment times (5.0 and 7.5 min) compared to the control. These results corroborate the importance of the sensory parameters of consistency and overall appearance, which were also significant in consumer purchase choices. The sensory analysis demonstrated that ultrasound-treated samples were consistently better rated by participants than the control sample.

Regarding the fatty acid profile, a variety of changes were observed during the storage period, indicating that ultrasound affects the lipid composition of the beverages. Sugar analysis revealed that ultrasound contributed to the reduction in sucrose in the treated samples, potentially favoring the growth of probiotic microorganisms. Thus, these results suggest that high-intensity ultrasound not only improves the physical and chemical properties of probiotic beverages but also has a positive impact on consumer acceptance.

Finally, it is important to highlight that the use of HIUS in this study was effective in stabilizing the probiotic beverages by reducing syneresis and improving sensory properties, as the probiotic viability tested in our preliminary study suggests that ultrasound treatments, particularly for intermediate times of 2.5 to 7.5 min, have a positive impact on the physical, chemical, and sensory properties of the beverages. The results indicate that high-intensity ultrasound may be a valuable tool in improving probiotic beverage stability and consumer acceptance.

## Figures and Tables

**Table 1:** Syneresis results of the fermented probiotic strawberry beverage on days 1, 14, and 28 expressed in percentage (%).

Sample/Day	Day 01	Day 14	Day 28
Control	63.427 ^Ad^ ± 0.933	68.813 ^Bc^ ± 0.557	68.587 ^Bc^ ± 0.100
U2.5	59.013 ^Abc^ ± 2.109	63.743 ^Bb^ ± 0.733	64.610 ^Bb^ ± 0.226
U5.0	57.097 ^Ab^ ± 0.725	62.367 ^Bab^ ± 0.286	62.997 ^Bab^ ± 0.600
U7.5	52.057 ^Aa^ ± 2.141	60.747 ^Ba^ ± 2.331	61.167 ^Ba^ ± 1.900
U10	61.337 ^Acd^ ± 0.749	64.000 ^Bb^ ± 0.536	65.073 ^Bb^ ± 0.885

Lowercase letter superscripts indicate differences between samples on the same day (vertical); uppercase letter superscripts indicate differences between samples on different days (horizontal); *p* < 0.05.

**Table 2:** Fatty acid composition (g/100 g) of the total fat in the fermented beverage (day 1 and 28).

Fatty Acids	Control		U2.5		U5.0		U7.5		U10	
	Day 01	Day 28	Day 01	Day 28	Day 01	Day 28	Day 01	Day 28	Day 01	Day 28
Caprylic Acid C8:0	1.060 ^Bc^ ± 0.006	0.535 ^Aa^ ± 0.006	0.925 ^Ba^ ± 0.006	0.615 ^Ab^ ± 0.006	1.645 ^Bd^ ± 0.006	0.825 ^Ad^ ± 0.006	1.705 ^Be^ ± 0.006	0.645 ^Ac^ ± 0.006	0.955 ^Bb^ ± 0.006	0.735 ^Ad^ ± 0.006
Capric Acid C10:0	3.140 ^Bc^ ± 0.017	1.595 ^Aa^ ± 0.006	2.290 ^Ba^ ± 0.000	2.125 ^Ac^ ± 0.006	4.345 ^Bd^ ± 0.006	2.110 ^Ac^ ± 0.012	3.865 ^Bd^ ± 0.006	1.860 ^Ab^ ± 0.012	2.415 ^Bb^ ± 0.006	2.215 ^Ad^ ± 0.006
Lauric Acid C12:0	3.240 ^Bc^ ± 0.006	2.465 ^Aa^ ± 0.006	2.980 ^Ba^ ± 0.000	2.555 ^Ab^ ± 0.006	5.415 ^Bd^ ± 0.006	2.905 ^Ad^ ± 0.006	5.555 ^Be^ ± 0.006	2.745 ^Ac^ ± 0.006	3.175 ^Bb^ ± 0.006	2.905 ^Ad^ ± 0.006
Myristic Acid C14:0	11.100 ^Bb^ ± 0.006	10.115 ^Ab^ ± 0.006	10.570 ^Aa^ ± 0.010	12.105 ^Be^ ± 0.006	17.530 ^Be^ ± 0.010	9.880 ^Aa^ ± 0.012	15.115 ^Bd^ ± 0.006	10.665 ^Ad^ ± 0.006	11.220 ^Bc^ ± 0.000	10.165 ^Ac^ ± 0.006
Methyl Tetradecanoate C15:2	0.610 ^Bb^ ± 0.006	0.555 ^Aa^ ± 0.006	0.625 ^Ac^ ± 0.010	0.635 ^Ad^ ± 0.006	0.615 ^Ab^ ± 0.006	0.605 ^Ac^ ± 0.006	0.590 ^Aa^ ± 0.000	0.605 ^Ac^ ± 0.006	0.590 ^Aa^ ± 0.000	0.585 ^Ab^ ± 0.006
Pentadecanoic Acid C15:0	1.510 ^Bd^ ± 0.012	1.055 ^Ab^ ± 0.006	1.055 ^Ab^ ± 0.006	1.035 ^Aa^ ± 0.006	1.015 ^Aa^ ± 0.006	1.065 ^Bb^ ± 0.006	1.105 ^Ac^ ± 0.006	1.101 ^Ac^ ± 0.000	1.095 ^Ac^ ± 0.006	1.205 ^Bd^ ± 0.006
Palmitic Acid C16:0	31.310 ^Ab^ ± 0.100	31.885 ^Ab^ ± 0.006	30.165 ^Aa^ ± 0.006	31.890 ^Bb^ ± 0.012	39.170 ^Be^ ± 0.010	31.925 ^Abc^ ± 0.006	35.645 ^Bd^ ± 0.006	31.975 ^Ac^ ± 0.006	31.645 ^Ac^ ± 0.006	31.135 ^Aa^ ± 0.006
Margaric Acid C17:0	0.710 ^Bb^ ± 0.006	0.675 ^Aa^ ± 0.005	0.675 ^Aa^ ± 0.006	0.735 ^Bb^ ± 0.006	0.775 ^Ac^ ± 0.006	0.805 ^Bc^ ± 0.006	0.685 ^Aa^ ± 0.006	0.815 ^Bc^ ± 0.006	0.705 ^Ab^ ± 0.006	0.725 ^Bb^ ± 0.006
Stearic Acid C18:0	12.310 ^Ad^ ± 0.005	14.195 ^Bd^ ± 0.006	11.740 ^Ac^ ± 0.012	14.285 ^Be^ ± 0.006	8.435 ^Ab^ ± 0.006	12.945 ^Ba^ ± 0.006	7.860 ^Aa^ ± 0.012	13.625 ^Bc^ ± 0.006	12.465 ^Ae^ ± 0.006	13.215 ^Bb^ ± 0.006
Oleic Acid C18:1	21.630 ^Ad^ ± 0.006	23.275 ^Be^ ± 0.006	19.775 ^Ac^ ± 0.006	20.635 ^Ba^ ± 0.006	15.335 ^Ab^ ± 0.006	21.980 ^Bc^ ± 0.012	14.040 ^Aa^ ± 0.012	22.435 ^Bd^ ± 0.006	22.340 ^Be^ ± 0.012	21.465 ^Ab^ ± 0.006
Elaidic Acid C18:1 trans-9	3.980 ^Ab^ ± 0.012	4.225 ^Bc^ ± 0.006	3.980 ^Ab^ ± 0.000	4.105 ^Bb^ ± 0.006	3.465 ^Aa^ ± 0.006	4.255 ^Bd^ ± 0.006	3.465 ^Aa^ ± 0.006	3.975 ^Ba^ ± 0.006	4.375 ^Bc^ ± 0.006	4.275 ^Ae^ ± 0.006
Palmitoleate Acid C16:1n7	1.760 ^Ae^ ± 0.006	1.620 ^Ab^ ± 0.010	1.575 ^Ac^ ± 0.006	1.525 ^Aa^ ± 0.006	1.115 ^Aa^ ± 0.006	1.660 ^Bc^ ± 0.012	1.440 ^Ab^ ± 0.012	1.725 ^Bd^ ± 0.006	1.725 ^Ad^ ± 0.006	1.665 ^Ac^ ± 0.006
Linolenic Acid C18:2n-3	0.380 ^Abc^ ± 0.006	0.345 ^Ab^ ± 0.021	0.345 ^Aa^ ± 0.006	0.365 ^Bb^ ± 0.006	0.365 ^Aab^ ± 0.006	0.365 ^Ab^ ± 0.006	0.395 ^Bc^ ± 0.006	0.305 ^Aa^ ± 0.006	0.375 ^BCb^ ± 0.006	0.350 ^Ab^ ± 0.000
Arachidonic Acid C20:4n-6	0.120 ^Aa^ ± 0.008	0.125 ^Aa^ ± 0.006	0.175 ^Aa^ ± 0.006	0.145 ^Aa^ ± 0.006	0.205 ^Aa^ ± 0.006	0.140 ^Aa^ ± 0.006	0.135 ^Aa^ ± 0.006	0.105 ^Aa^ ± 0.006	0.165 ^Aa^ ± 0.006	0.155 ^Aa^ ± 0.006
Linoleic Acid C18:2n-6	2.720 ^Bc^ ± 0.012	2.005 ^Aa^ ± 0.006	2.040 ^Aa^ ± 0.012	2.015 ^Bb^ ± 0.006	2.060 ^Ac^ ± 0.010	2.420 ^Bc^ ± 0.012	2.425 ^Aa^ ± 0.006	2.050 ^Bc^ ± 0.012	2.385 ^Ab^ ± 0.006	2.165 ^Bb^ ± 0.006
∑SFA	64.990 ^Bc^ ± 0.153	63.075 ^Ab^ ± 0.053	61.025 ^Ba^ ± 0.056	65.980 ^Ad^ ± 0.060	59.770 ^Ae^ ± 0.062	63.065 ^Bb^ ± 0.066	72.125 ^Bd^ ± 0.054	64.036 ^Ac^ ± 0.054	64.265 ^Bb^ ± 0.048	62.885 ^Aa^ ± 0.054
∑MUFA	27.340 ^Ab^ ± 0.024	29.120 ^Bc^ ± 0.022	25.330 ^Ab^ ± 0.012	26.260 ^Ab^ ± 0.018	19.915 ^Aa^ ± 0.018	27.895 ^Bbc^ ± 0.03	18.945 ^Aa^ ± 0.03	28.135 ^Bc^ ± 0.018	28.435 ^Ab^ ± 0.025	27.405 ^Abc^ ± 0.018
∑PUFA	3.220 ^Bc^ ± 0.030	2.780 ^Ab^ ± 0.033	2.560 ^Aa^ ± 0.024	2.525 ^Aab^ ± 0.018	2.630 ^Aa^ ± 0.022	2.925 ^Bd^ ± 0.024	2.955 ^Bb^ ± 0.018	2.460 ^Aa^ ± 0.024	2.925 ^Bb^ ± 0.018	2.670 ^Ac^ ± 0.012
∑n-6	2.840 ^Bc^ ± 0.020	2.125 ^Aa^ ± 0.012	2.215 ^Ba^ ± 0.018	2.160 ^Aa^ ± 0.012	2.265 ^Aa^ ± 0.016	2.560 ^Bc^ ± 0.018	2.550 ^Bb^ ± 0.012	2.155 ^Aa^ ± 0.018	2.545 ^Ab^ ± 0.012	2.320 ^Bb^ ± 0.012
∑n-3	0.380 ^Abc^ ± 0.060	0.345 ^Ab^ ± 0.021	0.345 ^Aa^ ± 0.006	0.365 ^Bb^ ± 0.006	0.365 ^Aab^ ± 0.006	0.365 ^Ab^ ± 0.006	0.395 ^Bc^ ± 0.006	0.305 ^Aa^ ± 0.006	0.375 ^BCb^ ± 0.006	0.350 ^Ab^ ± 0.000
∑n-6/∑n-3 *	7.474 ± 0.333	6.159 ± 0.572	6.420 ± 3.000	5.918 ± 2.000	6.205 ± 2.667	7.014 ± 3.000	6.456 ± 2.000	7.066 ± 3.000	6.787 ± 2.000	6.629 ± 0.012
∑PUFA/∑SFA *	0.0495 ± 0.196	0.044 ± 0.622	0.0420 ± 0.429	0.038 ± 0.300	0.044 ± 0.355	0.046 ± 0.364	0.041 ± 0.330	0.038 ± 0.444	0.045 ± 0.375	0.042 ± 0.222

Lowercase letters indicate differences between samples on the same day (horizontal); uppercase letters indicate differences in samples on different days (horizontal); *p* < 0.05. SFA = saturated fatty acids; MUFA = monounsaturated fatty acids; PUFA = polyunsaturated fatty acids; n-6: = omega-6 fatty acids; n-3 = omega-3 fatty acids; PUFA/SFA and n-6/n-3 fatty acid ratio. * ∑n-6/∑n-3 and ∑PUFA/∑SFA are descriptive ratios, and no statistical analysis was performed for these values.

**Table 3:** Quantification of sugars (g/100 mL) on days 01, 14, and 28.

SAMPLE	DAY	Fructose	Sucrose	Lactose
CONTROL	01	0.3 ^Ba^ ± 0.1	9.4 ^Cc^ ± 0.1	6.5 ^Bc^ ± 0.0
	14	0.3 ^Ba^ ± 0.1	8.9 ^Ab^ ± 0.1	6.4 ^Bc^ ± 0.0
	28	0.2 ^Aa^ ± 0.0	8.8 ^Ab^ ± 0.0	6.2 ^Ab^ ± 0.0
U2.5	01	0.3 ^Ba^ ± 0.1	9.0 ^Bb^ ± 0.1	6.4 ^Bbc^ ± 0.0
	14	0.3 ^Ba^ ± 0.1	8.6 ^Aa^ ± 0.1	6.3 ^Bbc^ ± 0.0
	28	0.2 ^Aa^ ± 0.0	8.6 ^Aa^ ± 0.0	5.9 ^Aa^ ± 0.0
U5.0	01	0.3 ^Ba^ ± 0.1	8.9 ^Ba^ ± 0.0	6.2 ^Aa^ ± 0.1
	14	0.3 ^Ba^ ± 0.1	8.8 ^Ab^ ± 0.0	6.2 ^Aab^ ± 0.1
	28	0.2 ^Aa^ ± 0.0	8.8 ^Ab^ ± 0.0	6.2 ^Ab^ ± 0.1
U7.5	01	0.3 ^Ba^ ± 0.1	8.9 ^Ba^ ± 0.0	6.4 ^Bbc^ ± 0.0
	14	0.3 ^Ba^ ± 0.1	8.6 ^Aa^ ± 0.0	6.1 ^Aa^ ± 0.0
	28	0.2 ^Aa^ ± 0.0	8.9 ^Bb^ ± 0.0	6.4 ^Bc^ ± 0.0
U10	01	0.3 ^Ba^ ± 0.1	8.8 ^Ba^ ± 0.1	6.3 ^Bab^ ± 0.0
	14	0.3 ^Ba^ ± 0.1	8.6 ^Aa^ ± 0.1	6.1 ^Aa^ ± 0.0
	28	0.2 ^Aa^ ± 0.0	8.8 ^Bb^ ± 0.1	6.2 ^ABb^ ± 0.0

Lowercase letter superscripts indicate differences between samples on the same day (vertical); uppercase letter superscripts indicate differences in samples on different days (horizontal); *p* < 0.05.

## Data Availability

The original contributions presented in the study are included in the article; further inquiries can be directed to the corresponding author.
